# Synergistic Effects of Celecoxib and Bupropion in a Model of Chronic Inflammation-Related Depression in Mice

**DOI:** 10.1371/journal.pone.0077227

**Published:** 2013-09-27

**Authors:** Izaque S. Maciel, Rodrigo B. M. Silva, Fernanda B. Morrone, João B. Calixto, Maria M. Campos

**Affiliations:** 1 Postgraduate Program in Medicine and Health Sciencies, Pontifícia Universidade Católica do Rio Grande do Sul, Porto Alegre/RS, Brazil; 2 Postgraduate Program in Cellular and Molecular Biology, Pontifícia Universidade Católica do Rio Grande do Sul, Porto Alegre/RS, Brazil; 3 Institute of Toxicology and Pharmacology, Pontifícia Universidade Católica do Rio Grande do Sul, Porto Alegre/RS, Brazil; 4 Department of Pharmacology, Universidade Federal de Santa Catarina, Campus Universitário, Universidade Federal de Santa Catarina, Florianópolis/SC, Brazil; 5 Faculty of Pharmacy, Pontifícia Universidade Católica do Rio Grande do Sul, Porto Alegre/RS, Brazil; 6 Faculty of Dentistry, Pontifícia Universidade Católica do Rio Grande do Sul, Porto Alegre/RS, Brazil; Emory University, United States of America

## Abstract

This study was aimed to characterize the depression-like behaviour in the classical model of chronic inflammation induced by Complete Freund’s Adjuvant (CFA). Male Swiss mice received an intraplantar (i.pl.) injection of CFA (50 µl/paw) or vehicle. Behavioural and inflammatory responses were measured at different time-points (1 to 4 weeks), and different pharmacological tools were tested. The brain levels of IL-1β and BDNF, or COX-2 expression were also determined. CFA elicited a time-dependent edema formation and mechanical allodynia, which was accompanied by a significant increase in the immobility time in the tail suspension (TST) or forced-swimming (FST) depression tests. Repeated administration of the antidepressants imipramine (10 mg/kg), fluoxetine (20 mg/kg) and bupropion (30 mg/kg) significantly reversed depression-like behaviour induced by CFA. Predictably, the anti-inflammatory drugs dexamethasone (0.5 mg/kg), indomethacin (10 mg/kg) and celecoxib (30 mg/kg) markedly reduced CFA-induced edema. The oral treatment with the analgesic drugs dipyrone (30 and 300 mg/kg) or pregabalin (30 mg/kg) significantly reversed the mechanical allodyinia induced by CFA. Otherwise, either dipyrone or pregabalin (both 30 mg/kg) did not significantly affect the paw edema or the depressive-like behaviour induced by CFA, whereas the oral treatment with dipyrone (300 mg/kg) was able to reduce the immobility time in TST. Noteworthy, CFA-induced edema was reduced by bupropion (30 mg/kg), and depression behaviour was prevented by celecoxib (30 mg/kg). The co-treatment with bupropion and celecoxib (3 mg/kg each) significantly inhibited both inflammation and depression elicited by CFA. The same combined treatment reduced the brain levels of IL-1β, as well as COX-2 immunopositivity, whilst it failed to affect the reduction of BDNF levels. We provide novel evidence on the relationship between chronic inflammation and depression, suggesting that combination of antidepressant and anti-inflammatory agents bupropion and celecoxib might represent an attractive therapeutic strategy for depression.

## Introduction

Major depression disorder (MDD) is a serious world-wide health problem, with a prevalence ranging from 4.4 to 18% of the population [1,2], being related to great expends in public health [3]. There are several theories regarding the pathogenesis of MDD and most studies suggest the involvement of environmental factors, associated with genetic and biochemical components [4,5]. Accumulated evidence has indicated a close relationship between the inflammatory processes and MDD [6,7]. Clinically, it is observed that patients under treatment with interferon-α (IFN-α) (to treat infectious diseases or cancer) develop symptoms of MDD [8]. Other studies corroborate these findings, indicating that patients with inflammatory chronic diseases (such as cardiovascular diseases, type-2 diabetes and rheumatoid arthritis) are more susceptible to present MDD [7,9]. Additionally, some studies have demonstrated that patients with MDD have higher circulating levels of pro-inflammatory cytokines [10]. These pieces of evidence clearly indicate a crosstalk between depression and chronic inflammation [11,12].

Animal studies show that injection of bacterial lipopolysaccharide (LPS) or pro-inflammatory cytokines elicits a condition described as sickness behaviour, characterized by decreased food consumption and locomotor activity, besides changes in the circadian cycle, which is followed by depressive behaviour [6,13]. In humans, the symptoms might include fever, nausea, anhedonia, irritation, and cognitive deficits. In fact, cytokine-induced depressive-like behaviour in animals has been well described before; the enhanced immobility appear to reflect helplessness in inescapable situations, such as in the forced-swimming (FST) and tail-suspension (TST) tests, an effect that is reversed by systemic treatment with clinically relevant antidepressant drugs [14,15]. Literature data has demonstrated a link between increased indoleamine 2,3, deoxygenase (IDO) activity and depressive-like behaviour in mice treated with bacterial products [16,17]. Additionally, our group reported that LPS-induced acute inflammatory process is accompanied by depressive-like behaviour in mice, an action which is reversed by selective kinin B_1_ receptor antagonists [15]. It has also been demonstrated that anti-inflammatory drugs, mainly selective cyclooxygenase-2 (COX-2) inhibitors, appear to contribute to the beneficial effects of antidepressant medicines, in either humans or animal models of depression [18,19]. Nevertheless, classical antidepressant drugs were found effective in reducing inflammatory parameters in LPS-stimulated microglial cells [20,21].

An elevated rate of patients with MDD diagnosis does not satisfactorily respond to the currently available antidepressant therapy. Additionally, many patients do not adhere to the existing therapy, because of the side effects of anti-depressants [22,23]. This study aimed to characterize the depressive-like behaviour in mice with chronic inflammation and nociception induced by Complete Freund’s Adjuvant (CFA). Our protocol was based on previous studies correlating CFA treatment and mood disorders [24]. As a first approach, we have tested the effects of several antidepressant and anti-inflammatory/analgesic drugs in our experimental paradigm. Secondly, we have made some efforts to better characterize the mechanisms implicated in chronic CFA inflammation-related depression.

## Materials and Methods

### Animals

Swiss male mice (25 to 30 g) were used in this study. Animals were housed under conditions of optimum light, temperature and humidity (12 h light-dark cycle, 22 ± 1 °C, under 60 to 80% humidity), with food and water provided *ad libitum*. Mice were obtained from Central animal house from the Universidade Federal de Pelotas (UFPEL, Brazil). Experiments were conducted in accordance with current guidelines for the care of laboratory animals and ethical guidelines for the investigation of experimental pain in conscious animals laid down by [25]. All the experimental procedures were approved by the Animal Ethics Committee of Pontifícia Universidade Católica do Rio Grande do Sul (RS) – CEUA 09/00104.

### Chronic inflammation induction and evaluation

A chronic inflammatory response was induced by a unique unilateral intraplantar injection of complete Freund’s adjuvant (CFA; 1 mg/ml; heat-killed and dried *Mycobacterium tuberculosis*, each milliliter of vehicle containing 0.85 ml paraffin oil plus 0.15 ml mannide mono-oleate; Sigma, St Louis, MO, USA), in a volume of 50 µl, into the plantar surface of the right hindpaw. Control animals received 50 µl of saline (0.9% NaCl solution). The CFA dose was selected on the basis of previous publications [17,24] At 1, 2, 3 and 4 weeks following CFA application, the paw edema volume (in ml) was measured by using a plesthysmometer (Ugo Basile). The evaluation of time-related effects of CFA effects was performed using independent groups of animals to avoid additional stress to mice. To distinguish the sickness behaviour from depressive-like behaviour, both the body weight and the temperature of animals were assessed at different time-points after CFA injection. Besides, mice were subjected to open-field test to verify the changes in locomotor activity. In most cases, the depression-like behaviour was evaluated by TST (the protocols are described in details below). All the behaviour experiments were performed by trained experimenters blind to the treatment groups.

### Body temperature assessment

The mouse colonic temperature was recorded using a commercially available thermometer (Pro-check®). After recording the initial colonic temperature (t = 0; °C), the body temperature was evaluated before and 1, 3, 5, 7, 9, 11, 13, 14 days after CFA injection. The values were expressed as the difference between the temperatures on the day of measurement, minus the temperature before the injection of CFA (basal values).

### Body weight

Mouse body weight was recorded (in g) using a digital balance (Urano®). The animals were weighted before and once a day during 14 days after CFA injection. The values were expressed as the difference between the initial weights minus the weight values on the day of measurement.

### Open-field test

To analyze the locomotor activity, the animals were evaluated in the open-field test 2 weeks post CFA-injection. The experiments were conducted in a sound-attenuated room, under low-intensity light. Mice were individually placed in the centre of an acrylic box (40 x 60 x 50 cm), with the floor divided into 9 squares. The number of squares crossed with the four paws was registered during a period of 6 min.

### Tail-suspension test

To assess the depression-like behaviour, we have employed the TST, according to the methodology originally described by [26]. At different time-points following CFA treatment (1, 2, 3 and 4 weeks), the animals were suspended 50 cm above the floor by means of an adhesive tape, placed approximately 1 cm from the tip of the tail. The time during which mice remained immobile was quantified in seconds during a period of 6 min.

### Forced swimming test

To confirm the depressive-like behaviour induced by CFA, the animals were subjected to the forced swimming test (FST). The methodology used was the same described by [27]. The FST was carried out in a cylinder (18.5 cm diameter, 25 cm height) filled with water to the height of 17 cm. Water was maintained at 23-25°C. Mice were placed into the water and immobility was defined as absence of all movement except motions required for keeping the mouse’s head above the water. The time during which mice remained immobile was quantified in seconds during a period of 6 min.

### Mechanical allodynia

The measurement of the mechanical paw withdrawal threshold was carried out using the up-down paradigm, as described previously by [28], with minor modifications [29]. Briefly, the mice were firstly acclimatized during 1 h in individual clear Plexiglas boxes on an elevated wire mesh platform to allow access to the plantar surface of the hind paws. Von Frey filaments of increasing stiffness (0.02–10 g) were applied to the hind paw plantar surface of the animals with a pressure high enough to bend the filament. The absence of a paw lifting after 5 s led to the use of the next filament with increased weight, whereas paw lifting indicated a positive response and led to the use of the next weaker filament. This paradigm continued for a total of 6 measurements, including the one before the first paw-lifting response had been made, or until 4 consecutive positive (assigned a score of 0.030) or 4 consecutive negative (assigned a score of 6.76) responses occurred. The 50% mechanical paw withdraw threshold response was then calculated from the resulting scores as described previously by [30]. The paw withdraw threshold was expressed in grams (g) and was evaluated before and 2 weeks after the CFA injection. A significant decrease in paw withdraw threshold compared to baseline values was considered as mechanical allodynia. Independent groups were used to analyse mechanical allodyinia, paw edema and depression-like behavior.

### Protocols of treatment

To verify the effects of anti-inflammatory/analgesic and antidepressant drugs on the inflammatory and behavioural responses elicited by CFA, the animals were treated with the following drugs: the tricyclic antidepressant imipramine (10 mg/kgmg/kg, p.o.); the selective serotonin reuptake inhibitor (SSRI) fluoxetine (20 mg/kg, p.o.); the preferential dopamine reuptake blocker bupropion (3, 15 or 30 mg/kg, p.o.); the glucocorticoid dexamethasone (0.5 mg/kg, s.c.); the selective COX-2 inhibitor celecoxib (3, 15 or 30 mg/kg, p.o.); the dual COX-1/2 blocker indomethacin (10 mg/kg, p.o.); the analgesic drugs dipyrone (30 or 300 mg/kg, p.o.) or pregabalin (30 mg/kg, p.o.); once a day for 7 days.

In some cases, the animals received a combined treatment, as described: bupropion (3 mg/kg, p.o.) plus celecoxib (3 mg/kg, p.o.); once a day for 7 days. In all experimental sets, the treatment was initiated at the 7^th^ day after CFA injection, and continued until the 14^th^ day. The last injection was performed 1 h prior to behavioural assessments or edema evaluation. Independent groups were used to analyse the effects of anti-inflammatory or antidepressant drugs on paw edema or depression-like behavior. The schedules of treatment were determined on the basis of previous literature or on pilot experiments [15,31,32,33,34].

### Measurement of IL-1β levels

Fourteen days after CFA injection and *in vivo* functional tests, the animals were euthanized. The whole brains, hippocampus and cortex were removed and rapidly frozen. These brain regions had been previously used in previous literature reports using pre-clinical models of depression [35,36]. IL-1β levels were determined by means of a standard sandwich ELISA technique (detection limits = 4.8 pg/ml; assay range = 12.5-800 pg/ml and (CV% = intra-assay precision sample 1 = 7.5; 2 = 4.6 and 3 = 3) and (CV% = inter-assay precision sample 1 = 8.4; 2 = 6.6 and 3 = 5.7); R&D Systems, USA). The tissues were placed in a PBS solution containing 0.05% Tween 20, 0.1 mM PMSF, 0.1 mM benzamethonium chloride, 10 mM EDTA, 2 µg/ml aprotinin A, and 0.5% BSA. The tissues were homogenized and centrifuged at 3,000 x g for 10 min, and the supernatant was employed for ELISA analysis.

### Measurement of BDNF levels by ELISA and protein extraction

Fourteen days after CFA injection and *in vivo* functional tests, the animals were euthanized. The methodology used was the same as previously described with minimal changes [37]. The cortex and hippocampus were dissected and rapidly frozen. Both tissues were homogenized in lysis buffer (NaCl 137 mM; glycerol 10%; Tris-HCl 20 mM pH 8.0) containing a cocktail of protease inhibitors (Sigma, St. Louis, MO, USA). The homogenate was centrifuged (5.600 g, 15 min), and the supernatant was removed and stored at -80°C. The BDNF levels were measured by ELISA (BDNF E_max_^®^ ImmunoAssay System kit; detection limits = 15.6 pg/ml and CV% = (low = 8.8); (medium = 2.9) and (high = 2.2); Promega, Madison, WI, USA).

### Immunohistochemistry for cyclooxygenase 2 (COX-2)

The expression of COX-2 was measured by immunohistochemistry, as previously described by [38]. The whole brains were rapidly excised 14 days after CFA injection and fixed in buffered neutral formalin. Sections of 4 µm were mounted onto gelatin-coated slides. Rabbit polyclonal antibody raised against COX-2 (1:1000; Santa Cruz Biotechnology, Santa Cruz, CA, USA) was diluted in Tris-buffered saline containing 0.3% Triton X-100, 2% donkey serum and 1% BSA, and the sections were incubated overnight at room temperature, before being incubated for 2 h with biotinylated donkey anti-rabbit antibody (1:1000; Amersham Pharmacia Biotech, Europe, Freiburg, Germany), for 2 h with avidin-biotin peroxidase complex (1:1000; Vectastain ABC kit, Vector laboratories, Burlingame, CA, USA), and finally revealed with diaminobenzidine via the nickel-enhanced glucose-oxidase method. The procedure also included negative controls with omission of the primary antibody, which did not show any immunoreactions. The images were captured by a digital camera (DS-5 M-L1, Nikon, NY, USA), connected to an optical microscope (Nikon Eclipse 50i), at 100-x magnification, and analyzed through the Image NIH ImageJ 1.36b Software. The number of COX-2 positive cells was quantified and expressed as the positive area per field. For this series of experiments, we have used four animals per group.

### Statistical analysis

The results are presented as the mean ± SEM. The percentages of inhibition were calculated as the mean of inhibitions obtained for each individual experiment. The statistical comparison was performed by one-way analysis of variance (ANOVA) or by two-way ANOVA, depending on the experimental protocol, followed by Bonferroni’s post-hoc test. For the comparisons of areas under the curve (AUC), unpaired Student’s *t* test was used. *P* values less than 0.05 (*p*<0.05) were considered as indicative of significance (GraphPad Prism 5.0, La Jolla, CA, USA).

### Drugs and reagents

The following drugs and reagents were used: imipramine, fluoxetine, bupropion, dexamethasone, indomethacin, celecoxib, dipyrone, pregabalin, PMSF, CFA, TMB, BSA, EDTA, benzamethonium chloride, aprotinin A and Tween-20, glycerol, Tris-HCl (all from Sigma Chemical Company, St. Louis, U.S.A).

## Results

### Depressive-like behaviour in mice with chronic inflammation induced by CFA

**Figure 1:**
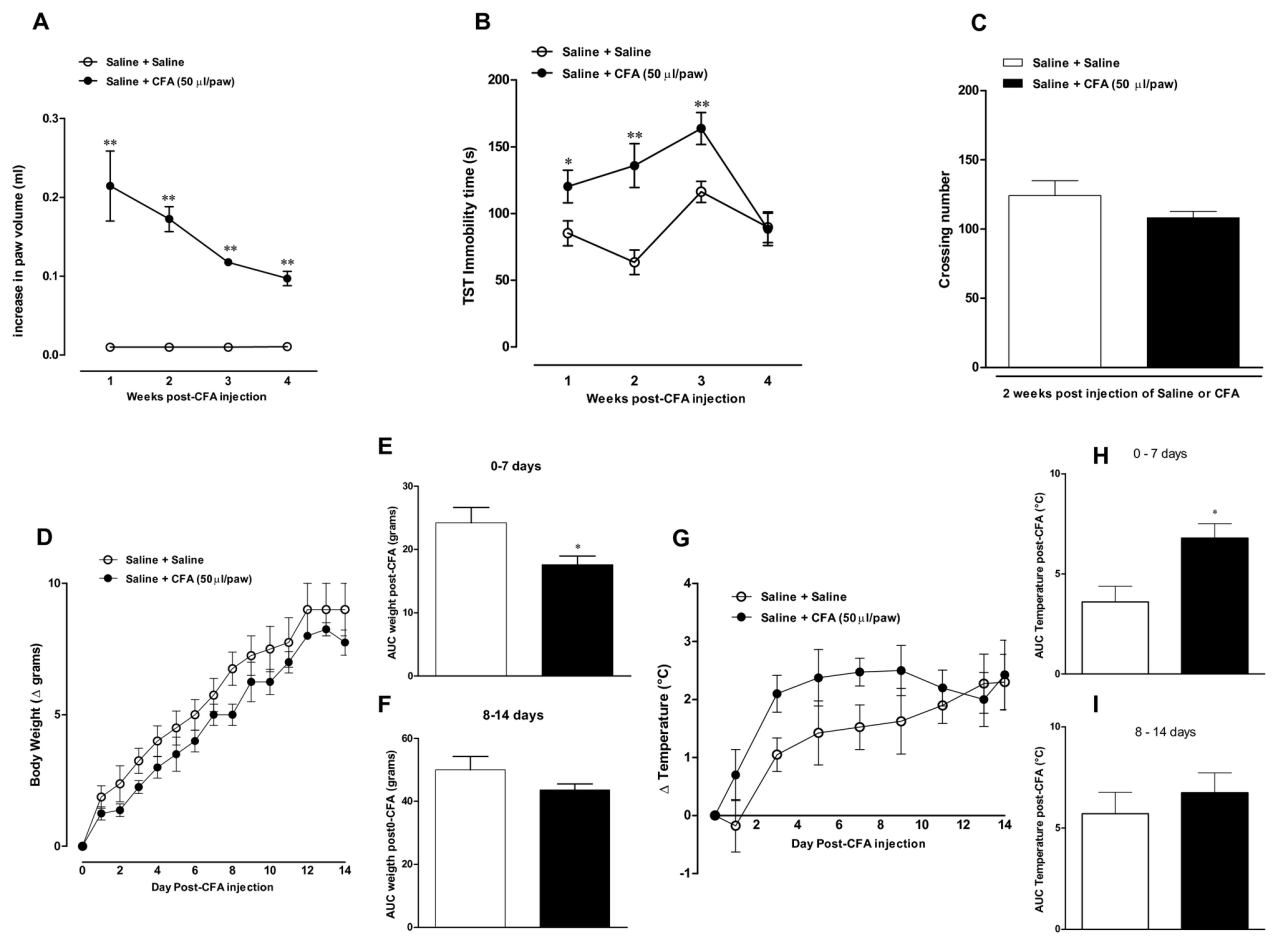
Time-related effects of CFA injection into the mouse paw. Effect of intraplantar injection of CFA (50 μl/paw) 1, 2, 3 and 4 weeks: (A) Paw edema analyzed in a plesthysmometer (difference between the right and the left paws), (B) immobility time in tail suspension test (TST), and (C) locomotor activity in the open-field test, (D) changes of body weight (Δ grams), (E) AUC of body weight, 0 to 7 days post-CFA injection, (F) AUC of body weight, 8 to 14 days post-CFA injection, (G) variation of body temperature, after injection of CFA (Δ °C), (H) AUC of body temperature 0 to 7 days post-CFA injection and (I) AUC of body temperature 8 to 14 days post-CFA injection. Each point or column represents the mean ± SEM of *6-8* animals per group. *P < 0.05 and **P < 0.01 significantly different from Saline+Saline group (ANOVA followed by Bonferroni’s post-hoc test).

This first experimental set was designed to characterize the relationship between inflammatory and behavioural changes following CFA injection. The intraplantar administration of CFA resulted in marked and time-dependent edematogenic response, which was maximal at one week ([1 week t=4.6, df=8; p<0.01; 2 weeks t=8.8, df=5; p<0.01; 3 weeks t=29.4, df=13; p<0.01 and 4 weeks t=8.5, df=13; p<0.01]; Figure 1A). Interestingly, the inflammatory response evoked by CFA was accompanied by a time-related increase in the immobility time, according to the assessment in the TST test ([41 ± 14% t=2.6, df=13; p<0.05; 74 ± 21% t=3.1, df=15; p<0.01 and 40 ± 10% t=3.2, df=25; p<0.01, at 1, 2 and 3 weeks, respectively]), that returned to the control values at 4 weeks (Figure 1B). Furthermore, our data revealed a decrease in body weight (Figure 1D and 1E) [t=2.3, df=8; p<0.05] and an increase in rectal temperature (Figure 1G and 1H) [t=2.9, df=8; p<0.05], at one-week period of evaluation. On the other hand, both parameters remained unaffected 2 weeks after CFA injection, when compared to control animals (Figure 1F and 1I). We also evaluated whether the reduction in immobility time might be related to locomotor deficits due to the arthritis development induced by CFA. Of note, the general locomotor activity of mice was not significantly altered at 2 weeks after CFA injection [t=1.464, df=6; p>0.05]; (Figure 1C). On the basis of our former results, we adopted the time-point of 2 weeks after CFA injection for the next experiments.

### Effects of treatment with antidepressant and anti-inflammatory drugs in the tail suspension test and paw edema formation

**Figure 2:**
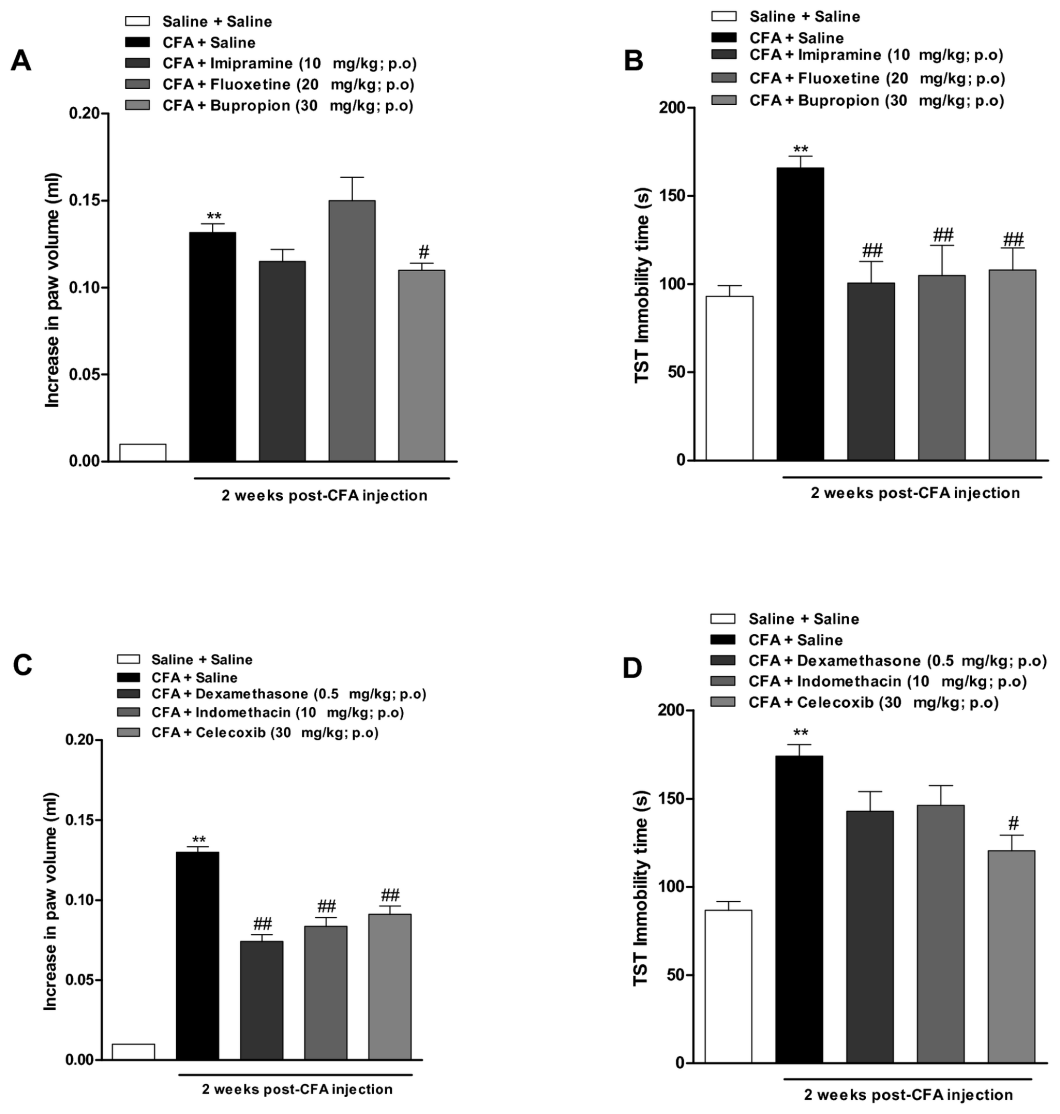
Evaluation of anti-inflammatory and antidepressants drugs in depressive-like behaviour and paw edema induced by CFA. Effect of intraplantar injection of CFA (50 μl/paw) at 2 weeks. (A and C) Paw edema analyzed in a plesthysmometer (difference between the right and the left paws), and (B and D) immobility time in tail suspension test (TST). Effect of treatment with imipramine, fluoxetine and bupropion (10 mg/kg, 20 mg/kgand 30 mg/kg, p.o, once a day, 7 days; respectively) or dexamethasone, indomethacin and celecoxib (0.5 mg/kg, s.c., 10 mg/kg, p.o. and 30 mg/kg, p.o., once a day, for 7 days, respectively). Independent groups were used to analyse the effects of anti-inflammatory or antidepressant drugs on paw edema or depression-like behavior. Each column represents mean ± SEM of *6-8* animals per group. *P < 0.05 and **P < 0.01 significantly different from Saline+Saline group; ^#^ P < 0.05 and ^# #^ P < 0.01 significantly different from CFA+Saline group (ANOVA followed by Bonferroni’s post-hoc test).

As a second approach, we evaluated a series of antidepressant and anti-inflammatory drugs in both the edema formation and the immobility time in TST, in 2-weeks CFA-treated animals. Figure 2B shows that CFA-elicited increase in immobility time was completely reversed to the basal levels, by treating animals (from the 7^th^ to the 14^th^ day) with the antidepressant drugs imipramine (10 mg/kg), fluoxetine (20 mg/kg) or bupropion (30 mg/kg), all given by oral route [F(4, 76) = 15.7; p<0.01]. Of note, the same treatment with bupropion, but not imipramine or fluoxetine, was able to significantly reduce the edema formation caused by CFA in 17 ± 4% [F(4, 70) = 154; p<0.05] (Figure 2A).

Data depicted in Figure 2C shows that repeated oral treatment with dexamethasone (0.5 mg/kg), the non-selective COX inhibitor indomethacin (10 mg/kg) or the selective COX-2 blocker celecoxib (30 mg/kg) produced a marked decrease of mouse paw edema induced by CFA, with inhibitions of 43 ± 3%, 36 ± 4% and 30 ± 4%, respectively [F(5, 110) = 200 p<0.01]. On the other hand, both dexamethasone and indomethacin failed to significantly affect the depressive-like behaviour in CFA-treated mice, whereas celecoxib visibly reversed this parameter (61 ± 10% [F(5, 73) = 25.0; p<0.05]; Figure 2D).

### Effects of the analgesic drugs dipyrone and pregabalin

**Figure 3:**
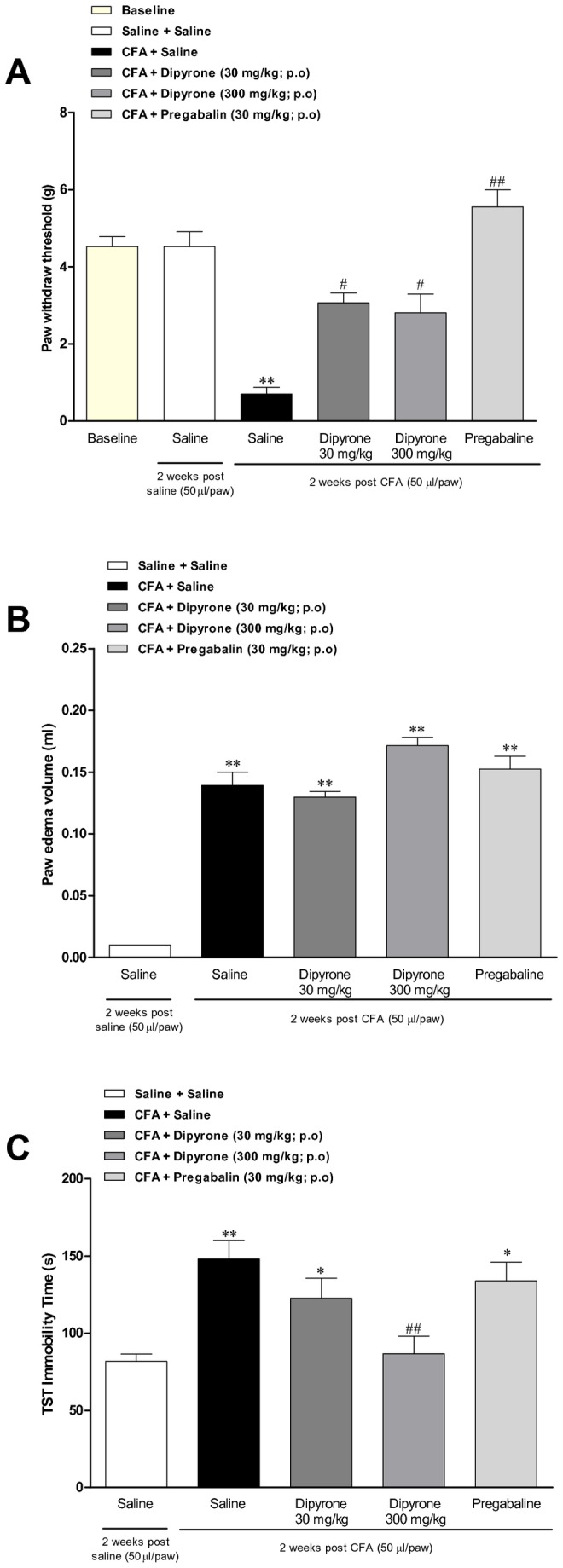
Assessment of pregabalin and dipyrone effects on the mechanical allodynia, paw edema and depressive-like behaviour induced by CFA. Effect of intraplantar injection of CFA (50 μl/paw) at 2 weeks. (A) Mechanical paw withdraw threshold analyzed by using the von Frey test; (B) Paw edema analyzed in a plesthysmometer (difference between the right and the left paws); and (C) immobility time in the tail suspension test (TST). Effects of treatment with dipyrone (30 and 300 mg/kg, p.o., once a day, during 7 days) or pregabalin (30 mg/kg, p.o., once a day, for 7 days). Each column represents the mean ± SEM of 6 to 8 animals per group. *P < 0.05 and **P < 0.01 significantly different from saline + saline group, and ^#^ P < 0.05 and ^# #^ P < 0.01 significantly different from CFA + saline group (ANOVA followed by Bonferroni’s post-hoc test).

We have also investigated whether depressive-like behaviour might be dependent on nociceptive alterations induced by CFA in our experimental paradigm. The results demonstrate that CFA injection induced mechanical allodynia, as characterized by a significant reduction in the paw withdraw threshold, according to assessment by using von Frey filaments (paw withdraw threshold diminished from 4.5 ± 0.4 g in baseline to 0.7 ± 0.3 g, at 2 weeks). As expected, the oral treatment with dipyrone (30 or 300 mg/kg, during 7 days) or pregabalin (30 mg/kg, during 7 days) significantly inhibited the mechanical allodyinia induced by CFA (paw withdraw threshold from 0,7 ± 0,3 g in the control group to 3.1 ± 0.2 g; 3.1 ± 0.4 g and 5.5 ± 0.45 g, respectively), in dipyrone- or pregabalin-treated animals [F(5, 92) = 18.5; p<0.01]; (Figure 3A). In contrast, either dipyrone (30 or 300 mg/kg, during 7 days) or pregabalin (30 mg/kg, during 7 days) did not significantly affect the paw edema formation induced by CFA [F(4, 44) = 60.67; p˃0.05]; (Figure 3B). However, the oral treatment with a higher dose of dipyrone (300 mg/kg, during 7 days) significantly reduced the TST immobility time in CFA-treated animals [F(4, 55) = 9.6; p<0.01], although this parameter was not affected by the administration of dipyrone or pregabalin (both 30 mg/kg; Figure 3C).

### Assessment of dose-related effects of bupropion and celecoxib

**Figure 4:**
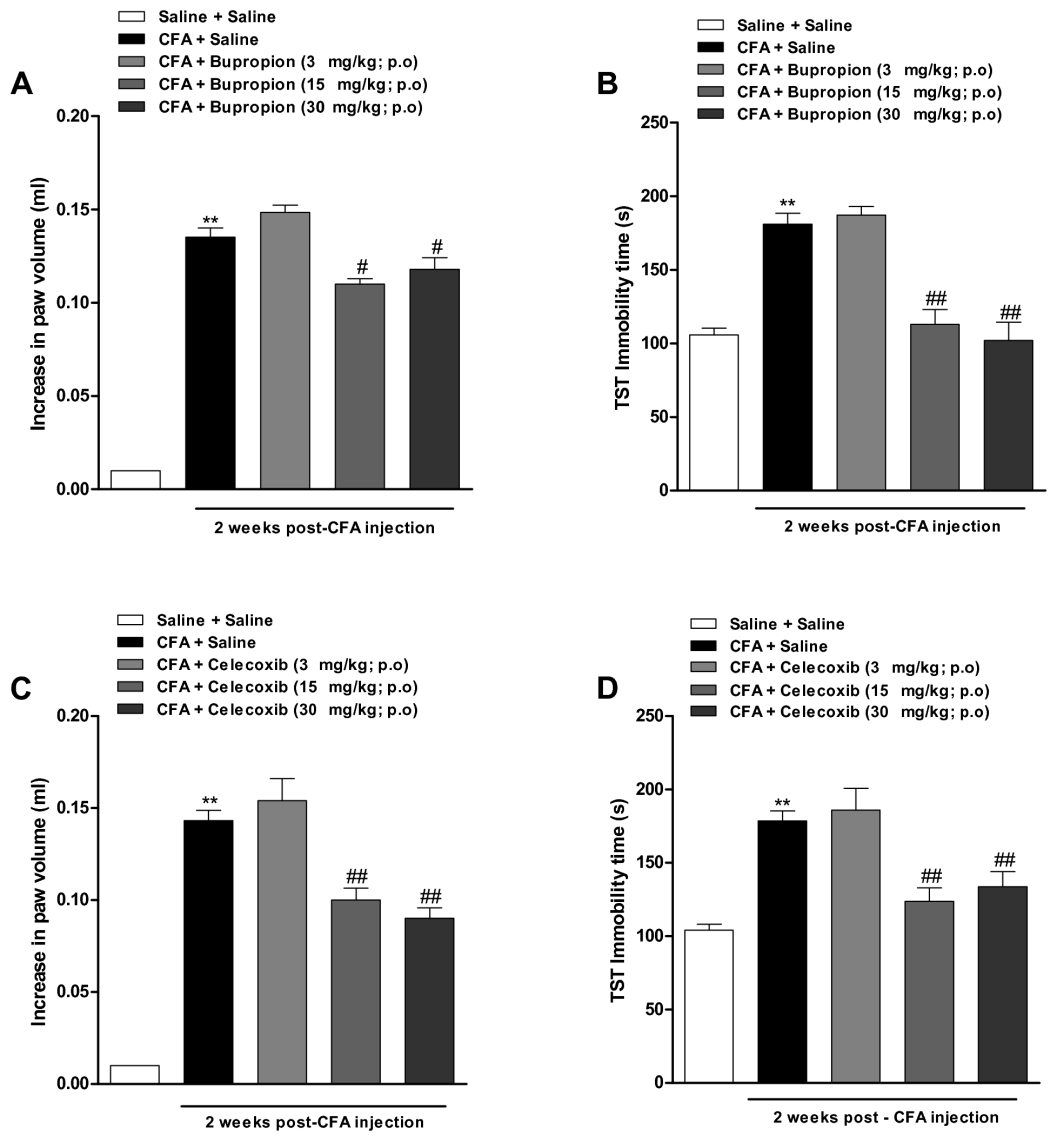
Dose-response analysis for the anti-inflammatory and antidepressant activities of celecoxib and bupropion. Effect of intraplantar injection of CFA (50 μl/paw) at 2 weeks. (A and C) Paw edema analyzed in a plesthysmometer (difference between the right and the left paws), and (B and D) immobility time in tail suspension test (TST). Dose-response effect of bupropion (3, 15 and 30 mg/kg, p.o., once a day, 7 days) or celecoxib (3, 15 and 30 mg/kg, p.o., once a day, 7 days). Independent groups were used to analyse the effects of anti-inflammatory or antidepressant drugs on paw edema or depression-like behavior. Each column represents the mean ± SEM of *6-8* animals per grup. **P < 0.01 significantly different from Saline+Saline group, ^#^ P < 0.05 and ^# #^ P < 0.01 significantly different from CFA+Saline gropu (ANOVA followed by Bonferroni’s post-hoc test).

We clearly demonstrated that either bupropion or celecoxib displayed significant anti-depressant and anti-inflammatory activities in our experimental paradigm. Therefore, we decided to conduct a dose-response experiment with both drugs. The results show that CFA-induced paw edema was significantly diminished by the oral treatment with bupropion and celecoxib, given at the doses of 15 and 30 mg/kg (with inhibitions of 20 ± 2% and 14 ± 5%; at 15 mg/kg and 30 mg/kg, respectively) [Figure 4A; F (4, 73) = 249.8; p<0.01] and [Figure 4C; F (4, 68) = 159.6; p<0.01]. Furthermore, bupropion and celecoxib, when administered at the doses of 15 and 30 mg/kg per oral route, were able to virtually reverse the depressive-like behaviour in TST [Figure 4B; F(4, 69) = 27.94; p<0.01] and [Figure 4D; F (4, 81) = 24.61; p<0.01]. Nevertheless, these parameters were not significantly altered by any drugs at 3 mg/kg. Therefore, this dose was selected for protocols designed to verify the effects of combined administration of celecoxib and bupropion.

### Synergistic interaction between bupropion and celecoxib

**Figure 5:**
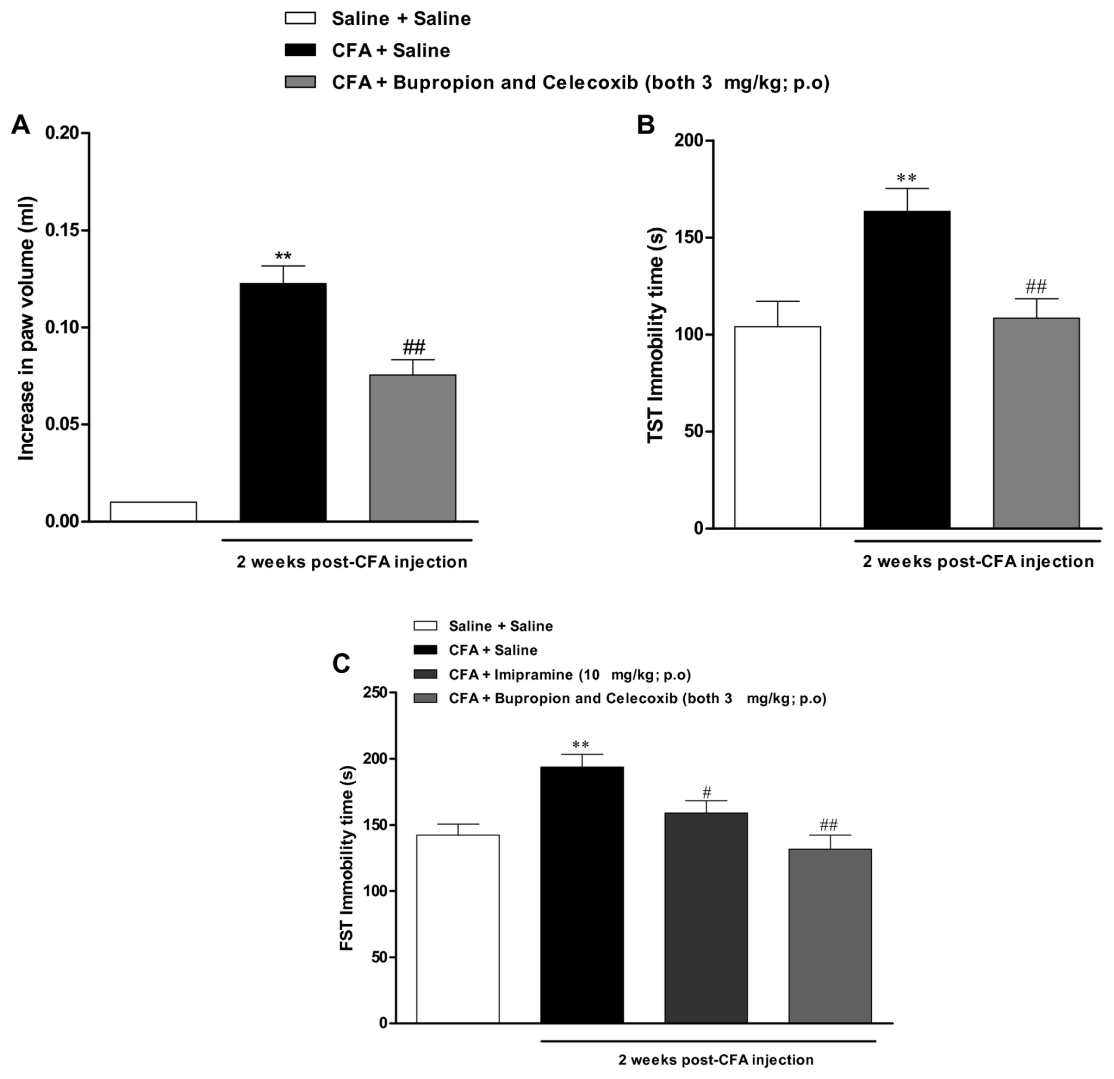
Effects of combined treatment with bupropion and celecoxib on CFA-induced inflammation and depression. Effect of intraplantar injection of CFA (50 μl/paw) at 2 weeks. (A) Paw edema analyzed in a plesthysmometer(difference between the right and the left paws), (B) immobility time in tail suspension test (TST), and (C) immobility time in forced swimming test (FST). Synergistic effect between different classes of drugs: bupropion and celecoxib both at (3 mg/kg, p.o., once a day, 7 days) and effect of treatment with imipramine (10 mg/kg, p.o., once a day, 7 days). Each column represents the mean ± SEM of *6-8* animals per group. **P < 0.01 significantly different from Saline+Saline group, ^#^ P < 0.05 and ^# #^ P < 0.01 significantly different from CFA+Saline group (ANOVA followed by Bonferroni’s post-hoc test).

Next, we decided to evaluate the effects of combined oral treatment with bupropion and celecoxib given at sub-therapeutic doses. Strikingly, the repeated administration of bupropion plus celecoxib (both at 3 mg/kg; once a day for 7 days) significantly reversed the depressive-like behaviour [Figure 5B; F (2, 22) = 7.9; p<0.01], as well as the paw edema formation [Figure 5A; F (2, 23) = 68.3; p<0.01] induced by CFA. The increase in immobility time was practically abolished by this combination, while the paw edema was reduced by 42 ± 6%.

To gain further insights on the effects of bupropion and celecoxib combination, we have also tested this strategy in another experimental assay of depression. The treatment with CFA resulted in a marked increase of immobility time in the FST, an effect that was significantly inhibited by the classical antidepressant imipramine (10 mg/kg, p.o.), administered from the 7^th^ to the 14^th^ day [Figure 5C; F (3, 33) = 8.7; p<0.01]. Interestingly, this depressive behaviour was markedly inhibited by the association sub-effective doses of bupropion and celecoxib, both administered at the dose of 3 mg/kg, confirming and extending the previous data on TST.

### IL-1β tracks with the depressive-like behaviour in mice with chronic inflammation induced by CFA

**Figure 6:**
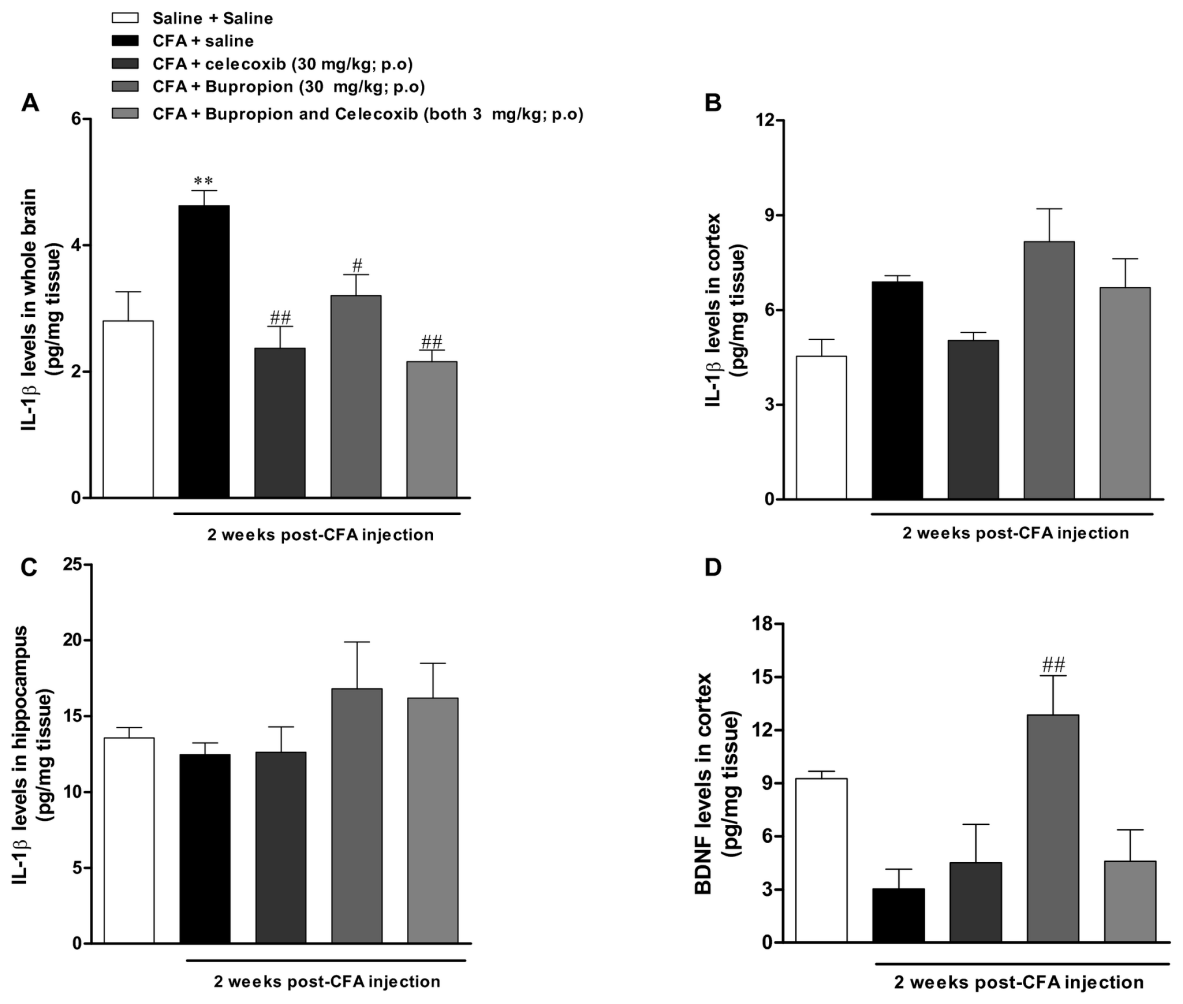
Determination of IL-1β, and BDNF levels in the brain of CFA-treated mice: effects of treatment with bupropion and celecoxib. Effect of intraplantar injection of CFA (50 μl/paw) at 2 weeks: IL-1β levels in the (A) whole brain, (B) cortex and (C) hippocampus. Effect of treatment with celecoxib and bupropion both at (30 mg/kg, p.o, once a day, 7 days) and combined effect of bupropion plus celecoxib (3 mg/kg, p.o, once a day, 7 days). (D) BDNF levels in cortex. Effects of treatment with celecoxib and bupropion (both at 30 mg/kg, p.o, once a day, 7 days) and combination of bupropion and celecoxib (both at 3 mg/kg, p.o, once a day, 7 days). Each column represents the mean ± SEM of *4* animals per group. **P < 0.01 significantly different from Saline+Saline group, ^#^ P < 0.05 and ^# #^ P < 0.01 significantly different from CFA+Saline group (ANOVA followed by Bonferroni’s post-hoc test).

There was a significant increase in the levels of IL-1β in the whole brain of CFA-injected mice at 2 weeks, a response significantly inhibited by the repeated treatment with bupropion or celecoxib (both 30 mg/kg). Of interest, the combined oral treatment with bupropion and celecoxib (each 3 mg/kg) significantly inhibited the increased IL-1β whole brain levels [Figure 6A; F (4, 61) = 8.3; p<0.01]). The evaluation of IL-1β levels in the cortex (Figure 6B) displayed a similar profile as observed in the whole brain, whereas no significant change was seen in the hippocampus (Figure 6C). We have also evaluated the levels of TNF-α and IL-10 in the whole brain of CFA-injected mice, but no significant difference was obtained (data not shown).

### The chronic inflammation induced by CFA injection reduced the BDNF levels in brain cortex of mice

The Figure 6D shows that CFA injection results in a significant decrease of BDNF levels in brain cortex, at 2 weeks. The long-term treatment with celecoxib (30 mg/kg; once a day for 7 days) or bupropion plus celecoxib (both at 3 mg/kg; once a day for 7 days) failed to interfere with reduced levels of BDNF. On the other hand, the repeated treatment with bupropion (30 mg/kg; once a day for 7 days) reversed the BDNF levels near to the basal values [Figure 6D; F (4, 13) = 5.2; p<0.01]. BDNF levels in hippocampus were not significantly different among the experimental groups (data not shown).

### Increased immunolabelling for COX-2 in brain cortex after CFA injection

**Figure 7:**
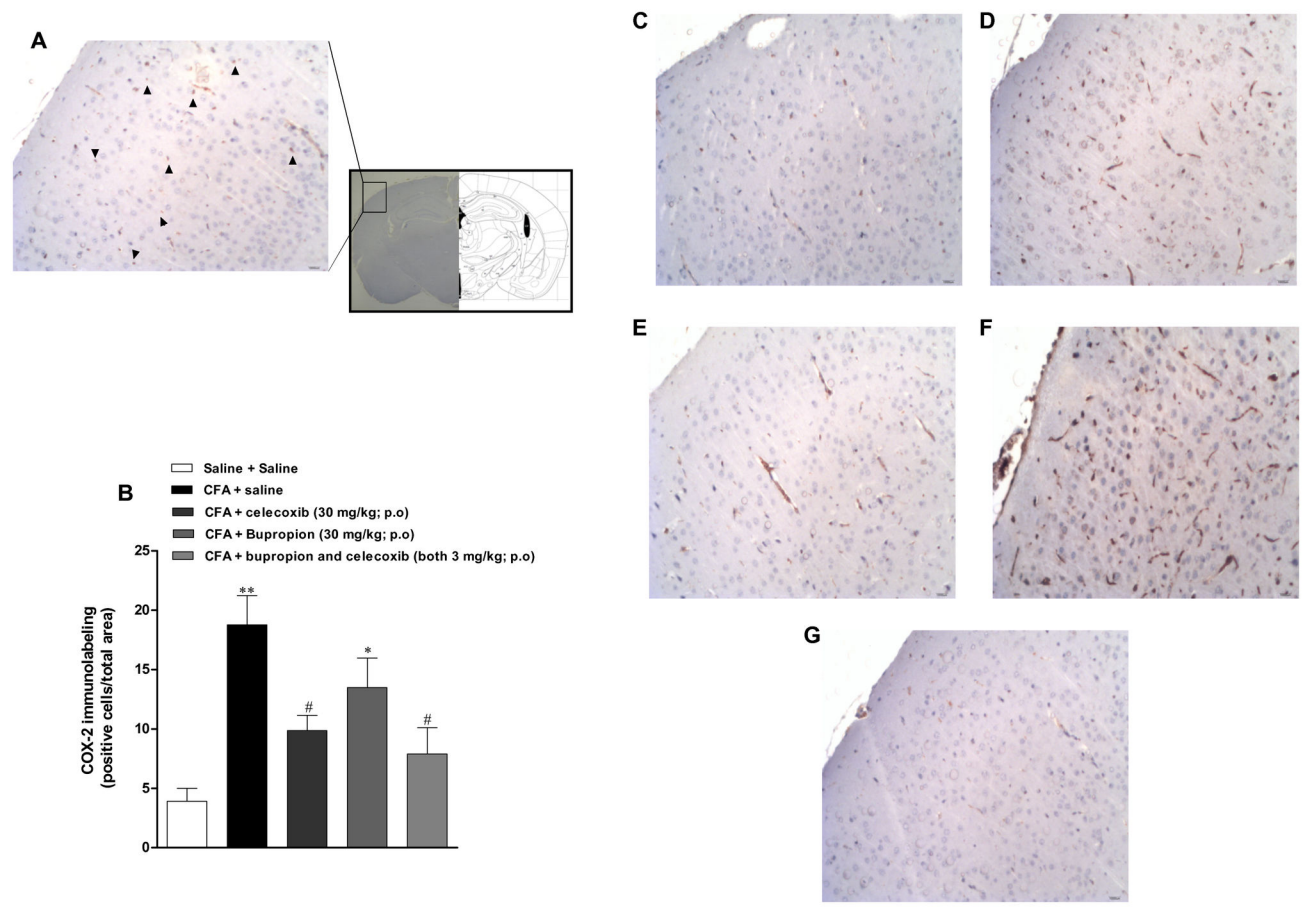
Immunolabelling for COX-2 in the cortex brain of CFA-treated mice: effects of treatment with bupropion and celecoxib. Immunohistochemistry analysis for COX-2 after intraplantar injection of CFA (50 μl/paw) at 2 weeks. Arrowheads (▲) indicate positive immunostaining for COX-2. Representative images of immunohistochemistry analysis for COX-2 in the mouse brain cortex: (A) schematic representation of the mouse brain indicating the region used for quantification (C) Control saline + saline group, (D) CFA + saline, (E) CFA + celecoxib (30 mg/kg, p.o., once a day, 7 days; respectively), (F) CFA + bupropion (30 mg/kg, p.o., once a day, 7 days; respectively), (G) CFA + bupropion and celecoxib (3 mg/kg, p.o., once a day, 7 days; both drugs). (B) Graphic showing the semi-quantitative analysis of immunostaining for COX-2. Scale bar represents 10,000 µm. Each column represents the mean ± SEM of *4* animals per group. **P < 0.01 significantly different from Saline+Saline group, ^#^ P < 0.05 significantly different from CFA+Saline group (ANOVA followed by Bonferroni’s post-hoc test).

The immunopositivity for COX-2 in CFA-injected mice was evaluated in the mouse cortex as depicted in the Figure 7A. At 2 weeks post-CFA injection, there was a significant increase in the immunolabelling for COX-2 in the CFA plus saline group, when compared to the control saline/saline group (Figure 7B, 7C and 7D). The repeated treatment with celecoxib (30 mg/kg) was able to significantly reduce the COX-2 immunolabelling, with an inhibition of 58 ± 9% (Figure 7B and 7E). However, the same treatment with bupropion (30 mg/kg) did not significantly affect COX-2 expression (Figure 7B and 7F). Interestingly, the long-term administration of bupropion plus celecoxib (both at 3 mg/kg; once a day for 7 days) markedly reduced the immunopositivity for COX-2, with an inhibition of 72 ± 15% (Figure 7B and 7G) [F(4, 37) = 6.9; p<0.01].

## Discussion and Conclusions

Data emerging from the present study provides convincing evidence that persistent inflammation induced by a single injection of CFA into the mouse paw is associated to development of depressive-like behaviour, as demonstrated by an increase in the immobility time in classical rodent models of depression. Moreover, this behaviour was largely prevented by the combined oral treatment with bupropion and celecoxib, when given at sub-therapeutic doses, as low as 3 mg/kg, by oral route. Importantly, our experimental evidence indicates that CFA-induced depressive state is likely independent on the inflammatory pain elicited by this phlogistic agent. Previous works have already demonstrated that inflammatory process (either acute or chronic) is associated to depression-like behaviour in animals [13,16]. Nevertheless, additional studies are still needed in order to further clarify the mechanisms linking inflammatory stimuli and depression.

Of note, signals indicative of sickness behaviour [16,39,40], namely the increase in body temperature and decrease in body weight, as well as the diminishment of locomotor activity in the open-field arena, were significantly changed only at the first week after CFA injection in our study. Considering that maximal changes in the immobility time in the TST were seen between 2 and 3 weeks after CFA injection, it is possible to discard the involvement of unspecific central effects in the depressive-like behaviour associated to CFA treatment. Furthermore, the treatment with the antidepressant drugs imipramine, fluoxetine and bupropion, once a day for 7 days, largely reversed the increase of immobility time in the TST, thereby corroborating the characterization of a depression model induced by chronic inflammation after CFA injection. This data confirms the growing body of evidence demonstrating that mood disorders, such as MDD, can be triggered by inflammation [13,41,42]. A comprehensive literature search revealed that fluoxetine, bupropion, or imipramine, when tested at a range of doses similar to that used in our study did not evoke any change of locomotor activity in the open-field test [43,44]. This might rather support our conclusions, on the basis of TST and FST experimental paradigm.

Chronic pain is commonly associated to behavioural changes [45,46], most likely by the activation of the hypothalamic-pituitary-adrenal (HPA) axis [47]. Of note, a recent study demonstrated that acute oral administration of high doses of the analgesic agent dipyrone displayed antidepressant-like effects in the mouse model of acute inflammation elicited by LPS [32]. Additionally, a recent publication showed a clear relationship between chronic pain and depression, in a rat model of inflammatory arthritis elicited by the intra-articular injection of CFA, via mechanisms involving the upregulation of IDO [17]. We wondered whether depressive-like behaviour might be dependent on nociceptive changes elicited by CFA in mice. There is a series of previous publications demonstrating that antidepressant drugs can display analgesic effects in different inflammation models, and some of them also show anti-inflammatory effects [21,48,49]. However, in the present study, we decided to evaluate dipyrone – considering its inability to interfere with inflammation; we also evaluated pregabalin – that represents a modern pharmacological alternative for treating chronic pain, even in mood-related situations, such as fibromyalgia [50]. Our results demonstrate that long-term administration of dipyrone, at 30 and 300 mg/kg, produced a marked inhibition of CFA-induced mechanical allodyinia, without affecting edema formation. Concerning the depressive-like behaviour, only the higher dose of dipyrone was able reduce the immobility time, according to assessment in TST. Therefore, we might suppose that nociception is not the prominent factor triggering depressive-like behaviour induced by CFA. This conclusion is further supported by data showing that the calcium channel blocker pregabalin, when administered at the analgesic dose of 30 mg/kg [31,33,34,51], effectively prevented the mechanical allodyinia induced by CFA, but failed to alter either the paw edema or the immobility time in our experimental paradigm. Higher doses of pregabalin were not tested in our study, as it has been demonstrated that elevated doses might induce marked changes of locomotor activity in rodents [52,53,54].

In this work, we have also evaluated the pharmacological activity of a series of anti-inflammatory drugs, namely dexamethasone, indomethacin, and celecoxib. All of them were able to reverse the paw edema induced by injection of CFA, confirming previous literature data [31,51]. Interestingly, the antidepressant drug bupropion also displayed a significant anti-edematogenic activity in the CFA model. In fact, it has been demonstrated before that bupropion inhibits TNF-α synthesis in a mouse model of LPS-induced inflammation [55]. Notably, the anti-inflammatory drug celecoxib was able to significantly decrease the immobility time in TST. This result corroborates previous data from either clinical trials or animal studies, indicating the relevance of COX-2 in depression [56,57,58]. In the clinics, it is usual to combine two or more drugs for the treatment of major depression [59]. A recent study conducted by Johanson et al. [19] demonstrated that combination of low doses of celecoxib to fluoxetine or reboxetine produced an increase of cortical 5-HT and noradrenaline output. Herein, we show for the first time that combined oral treatment with bupropion and celecoxib, when given at sub-therapeutic doses, was effective to reverse the paw edema formation and increased immobility time induced by CFA injection. Our results are suggestive of a possible synergistic interaction between bupropion and celecoxib, and this strategy might be reasonably tested in clinics.

In the present study, we aimed to characterize depression-like behavior in chronically inflamed mice, but also to evaluate alternative strategies to control depressive-like behavior under inflammation. Thus, we have selected two models of depression with recognized predictive validity, namely TST and FST. Furthermore, we attempted to evaluate whether CFA-induced nociception might affect depressive-like behavior, justifying our experimental design. Nonetheless, our study has some limitations and further studies are still required to extend the characterization of the model presented herein, possibly by using face validity models of depression, including voluntary wheel running and sucrose preference test, as carried out beforehand by Moreau et al. (2008) [40] using BCG inoculation.

We demonstrated that CFA injection led to a significant increase of IL-1β levels in the whole brain mouse. Literature data demonstrated that treatment with *E. coli* LPS induced a marked elevation of IL-1β mRNA expression in different regions of the rat brain [60]. However, it was not possible to detect significant changes in serum IL-1β levels among the groups (data not shown), or even when hippocampus or cortex were assessed separately. Relevantly, the repeated treatment with celecoxib or bupropion prevented the increase of IL-1β levels induced by CFA in the whole brain. Of note, Felger and Miller [61] suggested that during chronic inflammation, pro-inflammatory cytokines can induce persistent alterations of brain dopamine transmission, by affecting synthesis, packaging or release of this neurotransmitter, what might explain the effects of bupropion in our study. In our study, the brains were not perfused, and IL-1β might well be derived from the vasculature, and/or from the brain cells (for instance, microglia or astrocytes). Nevertheless, this is also a clear indication that peripheral chronic inflammation, as induced by CFA injection into the mouse paw, can lead to brain inflammation, even if this is mediated by the neighbour vasculature.

Accumulated evidence indicates that prostaglandin (PGE_2_) and COX-2 participate in the signalling of inflammatory processes, and they are likely implicated in neuronal death and inflammation-mediated cytotoxicity [62,63]. We show that injection of CFA markedly increased the immunolabelling for COX-2 whereas the repeated treatment with celecoxib or combined administration of bupropion plus celecoxib (both at 3 mg/kg, p.o.) was able to decrease COX-2 expression. However, at this moment we cannot discriminate whether COX-2 expression is increased in brain cells or in vasculature of mouse brain cortex. Our data are in accordance with a previous study showing an increase of COX-2 protein expression in a model of MDD induced by subcutaneous injection of clomipramine in newborn rats [64]. Of note, more recently, it has been demonstrated an overexpression of mRNA of the gene that encoding COX-2 and other inflammatory proteins, according to evaluation of peripheral blood cells collected from patients with recurrent episodes of depression [65]. Nevertheless, the chronic treatment with bupropion failed to significantly affect CFA-induced COX-2 brain expression. This led us to believe that distinct mechanisms mediate celecoxib and bupropion effects in our model of depressive-like behaviour.

Neurotrophins belong to a family of proteins related to growth and survival of cells, and neuronal plasticity [66]. BDNF has been identified as a crucial factor in the aetiology of depression [66,67]. Recent publications have demonstrated that BDNF levels are found significantly decreased in hippocampus and cortex obtained from depressive patients, post-mortem. The same is also true in animal models of depression [68,69]. In our study, we have provided evidence showing that BDNF levels were markedly reduced in the brain cortex, following 2 weeks of CFA injection. Noteworthy, the unique pharmacological strategy tested by us that was able to reverse decreased expression of BDNF was bupropion (30 mg/kg, 7 days of treatment). This result confirms and extends previous data showing that antidepressant drugs increase BDNF levels [70]. It is possible to infer that BDNF modulation might well be one of the mechanisms of action of bupropion.

In conclusion, the present work provides novel evidence on the relevance of persistent inflammation as a triggering factor for depressive-like behaviour. Bupropion and celecoxib appear to act in a synergistic manner in preventing the depression in CFA-induced inflammation in mice. We propose that this combination might represent an interesting therapeutic alternative for the treatment of depression, especially in patients with chronic inflammatory diseases.
